# Knockdown of circ_0011946 targets miR-216a-5p/BCL2L2 axis to regulate proliferation, migration, invasion and apoptosis of oral squamous cell carcinoma cells

**DOI:** 10.1186/s12885-021-08779-4

**Published:** 2021-10-07

**Authors:** Ying Zhou, Shuhong Zhang, Zhonghan Min, Zhongwei Yu, Huaiwei Zhang, Jiao Jiao

**Affiliations:** 1 Department of Dentistry, Jiaxing Hospital of Traditional Chinese Medicine, Jiaxing University, No. 1501 Zhongshan East Road, Jiaxing, Zhejiang, 314001 China; 2 Department of Orthopaedics, Jiaxing Hospital of Traditional Chinese Medicine, Jiaxing University, No. 1501 Zhongshan East Road, Jiaxing, Zhejiang, 314001 China

**Keywords:** Oral squamous cell carcinoma, circ_0011946, miR-216a-5p, BCL2L2

## Abstract

**Background:**

Circular RNAs (circRNAs) are implicated in the development of oral squamous cell carcinoma (OSCC). The aim of current research is to elucidate the role and mechanism of circ_0011946 in the functional behaviors of OSCC cells.

**Methods:**

Circ_0011946, microRNA (miR)-216a-5p, B cell lymphoma-2-like 2 protein (BCL2L2) abundances were exposed by quantitative reverse transcription polymerase chain reaction (qRT-PCR) or western blot. Cell proliferation, migration, invasion and apoptosis were detected by MTT, colony formation assay, transwell, wound-healing and flow cytometry assays, respectively. Target correlation was tested by dual-luciferase reporter and RNA pull-down assays. An in vivo xenograft experiment was employed to investigate the function of circ_0011946 on tumor growth in vivo.

**Results:**

Circ_0011946 and BCL2L2 levels were increased, while miR-216a-5p level was decreased in OSCC tissues and cells. Circ_0011946 knockdown impeded proliferation, migration, and invasion, but promoted apoptosis in OSCC cells. Circ_0011946 functioned as a sponge for miR-216a-5p, and BCL2L2 was targeted by miR-216a-5p. Besides, miR-216a-5p or BCL2L2 knockdown partly attenuated the inhibitory influences of circ_0011946 silence or miR-216a-5p overexpression on OSCC cell progression. Furthermore, circ_0011946 post-transcriptionally regulated BCL2L2 through sponging miR-216a-5p. Moreover, circ_0011946 knockdown constrained OSCC tumor growth in vivo.

**Conclusion:**

Circ_0011946 silence repressed OSCC cell proliferation, migration, and invasion, but promoted apoptosis through the regulation of the miR-216a-5p/BCL2L2 axis.

**Supplementary Information:**

The online version contains supplementary material available at 10.1186/s12885-021-08779-4.

## Background

Oral cancer is a common oral disease, and the major risk factors are tobacco use and alcohol consumption [1]. Oral squamous cell carcinoma (OSCC) is one of the primary cases of oral cancer, with low survival rate and high incidence [2]. Exploring the pathogenesis of OSCC may support a new therapeutic strategy for OSCC.

Noncoding RNAs are related to OSCC progression [3]. Circular RNAs (circRNAs) with covalently closed structure [4], which have been implicated in the development of various cancers, such as OSCC [5, 6]. For instance, circ_0000140 can inhibit cell proliferation, metastasis by mediating miR-31/Hippo pathway in OSCC [7]. Circ_002178 can promote cell proliferation and migration by activating the protein kinase B/mammalian target of rapamycin (AKT/mTOR) pathway in OSCC [8]. CircRNA hsa_circ_0011946 (circ_0011946), derived from the back-splicing of exons of Scm polycomb group protein homolog 1 (SCMH1), is capacity of promoting OSCC cell development by regulating proliferating cells nuclear antigen (PCNA) [9]. Nevertheless, our understanding of the mechanisms of circ_0011946 regulation in OSCC is still limited.

MicroRNAs (miRNAs) are another type of small noncoding RNA molecules, and dysregulated miRNAs are associated with OSCC progression [10, 11]. A previous study uncovered that miR-216a-5p can repress the malignant phenotypes of OSCC via regulating eukaryotic translation initiation factor 4B (EIF4B) [12]. B cell lymphoma-2-like 2 protein (BCL2L2) belongs to the BCL2 family that exerts an important role in human cancers [13]. Moreover, BCL2L2 constrained the tumorigenesis of human cancers, like hepatocellular carcinoma and non-small cell lung cancer [14, 15]. More importantly, BCL2L2 can promote tumor development by increasing cell proliferation in OSCC [16]. However, whether miR-216a-5p and BCL2L2 participate in the regulation of circ_0011946 in OSCC development is undiscovered.

In present research, we scrutinized the influences and mechanism of circ_0011946 in the malignant phenotypes of OSCC cells.

## Methods

### Tissues collection

OSCC tumor tissues and paired adjacent normal samples (> 2 cm from tumor tissue) were collected 30 OSCC patients, who undergo surgery between 2012 and 2015 at Jiaxing Hospital of Traditional Chinese Medicine, Jiaxing University. Patients received any other therapy before surgery were excluded. All tissues were stored in liquid nitrogen. The clinical-parameters of OSCC patients were provided in Table 1. The research got the permission of the ethics committee of Jiaxing Hospital of Traditional Chinese Medicine, Jiaxing University with the obtained written informed consent.

**Table 1:** The clinicopathological parameters of patients with OSCC (n = 30)

Parameter	Case	Circ_0011946 expression	
		Low(*n* = 14)	High(*n* = 16)
Age (years)			
≤ 60	15	6	9
> 60	15	8	7
Sex			
Female	13	7	6
Male	17	7	10
Tumor size			
≤ 5 cm	16	11	5
> 5 cm	14	3	11
TNM stages			
I-II	14	9	5
III-IV	16	5	11

### Cell culture and transfection

OSCC cell lines (CAL27 and SCC25) were procured from Procell (Wuhan, China) and the culture medium is comprised of Dulbecco Modified Eagle’s medium (DMEM; Gibco, Gran Island, NY, USA) and 10% fetal bovine serum (Gibco). Human oral keratinocytes (HOK) were provided by Sciencell (Carlsbad, CA, USA) and cultured in oral keratinocyte medium (Sciencell) plus 1% oral keratinocyte growth supplement (Sciencell) and 1% antibiotic solution (Sciencell). All cells were cultivated at 37 °C in an incubator with 5% CO_2_.

BCL2L2 overexpression vector (pc-BCL2L2) was constructed by inserting human BCL2L2 coding sequence (lacking the 3’UTR) into pcDNA3.1 vector (HonorGene, Changsha, China), and empty vector was regarded as negative control (pc-NC). Small interfering RNA (si) of circ_0011946, miR-216a-5p mimic, miR-216a-5p inhibitor, and their negative controls (si-NC, miRNA NC and inhibitor NC) were procured from GenePharma (Shanghai, China), and mentioned in Table 2 CAL27 and SCC25 cells (1 × 10^5^) were introduced with 200 ng of vectors or/and 30 nM of miRNA mimic or/and 30 nM of miRNA inhibitor or/and 50 nM of siRNA using Liposomal Transfection reagent (Yeasen, Shanghai, China). Transfection efficacy was analyzed after 24 h.

**Table 2:** Sequences of oligonucleotides and primers used in this research

Name	Sequences (5′-3′)	
si-circ_0011946#1		5′-AGCACUAGAUGCUUUGGUGCC-3′
si-circ_0011946#2		5′-UUGCUCUAGAUGCUUUGGUGC-3′
si-circ_0011946#3		5′-CUAGAUGCUUUGGUGCCAGGA-3′
si-NC		5′-AACAGTCGCGTTTGCGACTGG-3′
miR-216a-5p mimic		5′-UAAUCUCAGCUGGCAACUGUGA-3’
miRNA NC		5′-UUCUCCGAACGUGUCACGU-3’
miR-216a-5p inhibitor		5′-UCACAGUUGCCAGCUGAGAUUA-3’
inhibitor NC		5′-CUAACGCAUGCACAGUCGUACG-3’
circ_0011946	sense	5′-TTCGACTCCCGAGACATCTT-3’
	antisense	5′-TCCTCTGTAGTGGAGCAGCA-3’
SCMH1	sense	5′-TCGTGGTGCAAACCTCTACC-3’
	antisense	5′-ACAGGGATCCTTCTCCTCCC-3’
BCL2L2	sense	5′-GGCAAGAACTAGGGGCAGTT-3’
	antisense	5′-ATGCACAAGGAAGGGGGATG-3’
miR-216a-5p	sense	5′-GCCGAGGTAATCTCAGCTGG-3’
	antisense	5′-CAGTGCGTGTCGTGGAGT-3’
U6	sense	5′-CTCGCTTCGGCAGCACA-3’
	antisense	5′-AACGCTTCACGAATTTGCGT-3’
GAPDH	sense	5′-GACAGTCAGCCGCATCTTCT-3’
	antisense	5′-GCGCCCAATACGACCAAATC-3’

### Quantitative reverse transcription PCR (qRT-PCR)

RNA was lysed using Trizol (Applygen, Beijing, China). Nuclear and cytoplasmic RNA were prepared via the Cytoplasmic & Nuclear RNA Purification Kit based on the manufacturer’s suggestion (Norgen Biotek, Thorold, ON, Canada). 1 μg RNA was exposed to reverse transcription with specific Reverse Transcriptase kit (iGeneBio, Guangzhou, China). The synthesized cDNA with SYBR (Toyobo, Osaka, Japan) and primers (Table 3) (Genscript, Nanjing, China) was used for qRT-PCR assay. The primer sequences were displayed in Table 2. U6 or GAPDH served as an internal control. Relative RNA level was decided by the 2^-ΔΔCt^ method [17], with U6 or GAPDH as an interior control.

### Actinomycin D and RNase R treatment

For the stability of circ_0011946, OSCC cells were stimulated with Actinomycin D (2 μg/mL; Cell Signaling Technology, Danvers, MA, USA) for 0, 4, 8, 12 or 24 h. For RNase R treatment, RNA (1 μg) was incubated with 2 U of RNase R (NovoBiotechnology, Beijing, China) at 37 °C for 20 min. Then, RNA was extracted, and circ_0011946, SCMH1 and GAPDH levels were uncovered by qRT-PCR.

### 3-(4,5-dimethylthiazol-2-yl)-2,5-diphenyltetrazolium bromide (MTT)

1 × 10^4^ cells/well CAL27 and SCC25 cells were inoculated onto the 96-well plates for 12 h. Next, 10 μL of MTT (0.5 mg/mL, Beyotime, Shanghai, China) was supplemented into per well. 4 h later, medium was replaced by 100 μL of dimethyl sulfoxide (Thermo Fisher Scientific, Waltham, MA, USA). The absorbance was assessed using a microplate reader (Allsheng, Hangzhou, China) at 570 nm.

### Colony formation assay

Briefly, 200 cells were inoculated into the 6-well plates for 10 days, and were spotted with 0.1% crystal violet (Solarbio, Beijing, China). The images were photographed, and the number of colonies (> 50 cells) was computed.

### Transwell and wound healing analyses

Cell invasion was analyzed with Matrigel-coated transwell chambers (Costar, Corning, NY, USA). Cell migration was tested using transwell chambers without Matrigel and wound healing assay. For transwell assay, CAL27 and SCC25 cells (1 × 10^4^ for migration assay, 3 × 10^4^ for invasion analysis) in serum-free DMEM were plated into the superior chambers, whereas the lower chamber was supplemented with 500 μL DMEM with 10% serum. 24 h upon incubation, migrated or invasive cells were spotted with 0.1% crystal violet, and examined under a microscope (× 100 magnification, Nikon, Tokyo, Japan) with at least 6 random fields.

For wound healing assay, 2 × 10^5^ CAL27 and SCC25 cells after various transfections were placed into each well of 6-well plates until 80% of confluency was reached. Next, a “wound” was made using a 200 μL pipette tip, and cells were allowed to migrate for additional 24 h. The images (× 100 magnification) at 0 and 24 h were recorded. Relative migratory rate was calculated by the formula: (si-circ_0011946_(wound area)0h_)-(si-circ_0011946_(wound area)24h_)/(si-NC_(wound area)0h_)-(si-NC_(wound area)24h_).

### Flow cytometry

Annexin V-FITC/Propidium Iodide (PI) apoptosis detection kit (Vazyme, Nanjing, China) was exploited for cell apoptosis analysis. A total of 2 × 10^5^ of transfected CAL27 and SCC25 cells was spotted with Annexin V-FITC and PI solution. Subsequently, cell apoptotic rate (Annexin V^+^/PI^−^ and Annexin V^+^/PI^+^) were recognized by a flow cytometer (Jiyuan Biotech, Guangzhou, China). .

### Western blot

Protein was detached in RIPA buffer (Applygen) and quantitated by a protein quantitative kit (Beyotime). 20 μg/lane Protein was segregated by sodium dodecyl sulfate-polyacrylamide gel and then transferred on polyvinylidene fluoride membrane (Solarbio). The membrane was blockaded in 5% non-fat milk, and then nurtured with the antibody against BCL2L2 (ab190952, 1:2000, Abcam, Cambridge, MA, USA) overnight and a secondary antibody IgG labeled by horseradish peroxidase (HRP) (ab97200, 1:20,000, Abcam) for 2 h. GAPDH (ab245356, 1:5000, Abcam) was used as a loading control. After hatching with ECL reagent (Amyjet Scientific, Wuhan, China), the blots were visualized by ChemiDoc XRS+ system (Bio-rad Laboratories, Richmond, California, USA) and analyzed Image J v1.8 software (NIH, Bethesda, MD, USA).

### Dual-luciferase reporter and RNA pull-down assays

The miRNA-binding sites to circ_0011946 and human 3’UTRs was predicted by starBase database. The segment of circ_0011946 or BCL2L2 3’UTR harboring the predicted or mutant miR-216a-5p binding fragments were subcloned into the pmirGLO vector (Promega, Madison, WI, USA) to construct the luciferase report vector WT-circ_0011946, WT-BCL2L2–3’UTR, MUT-circ_0011946 or MUT-BCL2L2–3’UTR. These luciferase reporter vectors (200 ng) together with miR-216a-5p mimic or miRNA NC (30 nM) were transfected into CAL27 cells (1 × 10^5^), respectively. 48 h upon incubation, the ratio of *firefly* to *Renilla* luciferase was estimated by the Dual-luciferase reporter assay (Promega).

RNA pull-down assay was implemented through Magnetic RNA pull-down kit (Thermo Fisher Scientific). 1 × 10^7^ CAL27 cells were lysed and hatched with the beads-conjugated biotin-labeled circ_0011946 probe or oligo probe (negative control) overnight at 4 °C. RNA bound to beads was isolated, and circ_0011946 and miR-216a-5p abundances were discovered by qRT-PCR.

### Xenograft experiment

BALB/c nude mice (male, 5-week-old) were procured from Beijing Laboratory Animal Center (Beijing, China) and fed in specific pathogen-free microisolator cages. Lentiviruses expressing shRNA-circ_0011946 (sh-circ_0011946) and the negative control (sh-NC) were supplied by Ribobio (Guangzhou, China). Stable CAL27 cells were established by transducing with sh-circ_0011946 or sh-NC, and the selection of puromycin. Stable CAL27 cells (3 × 10^6^/mouse) were hypodermically inoculated into the light flanks of the mice (*n* = 6/group). Tumor volume was estimated every week based on the formula: length × width^2^/2. 4 weeks later, all mice were euthanized using 5% isoflurane. Tumor tissues were collected and weighed. circ_0011946, miR-216a-5p and BCL2L2 levels in tumor samples were measured. Proliferation of the tumors was assessed with paraffin-embedded tumor tissues by immunohistochemistry under the standard method using the anti-Ki67 antibody (ab15580, 1:100 dilution, Abcam), biotinylated goat anti-rabbit IgG secondary antibody (ab64256, 1:300 dilution, Abcam) and a 3,3-diaminobenzidine (DAB) Kit (Vector Laboratories, Peterborough, UK). The animal experiments got the approval of the Institutional Animal Care and Use Committee of Jiaxing Hospital of Traditional Chinese Medicine, Jiaxing University.

### Statistical analysis

Data of three repetitions were represented as mean ± standard deviation (SD) through GraphPad Prism 7 (GraphPad Inc., La Jolla, CA, USA). Student *t*-test or ANOVA followed by Tukey test was exploited to analyze the difference. The Pearson’s rank correlation coefficient was exploited to ascertain the correlation of genes in OSCC tissues. *P* < 0.05 was deemed as statistical significant.

## Results

### Circ_0011946 abundance is elevated in OSCC

**Fig. 1:**
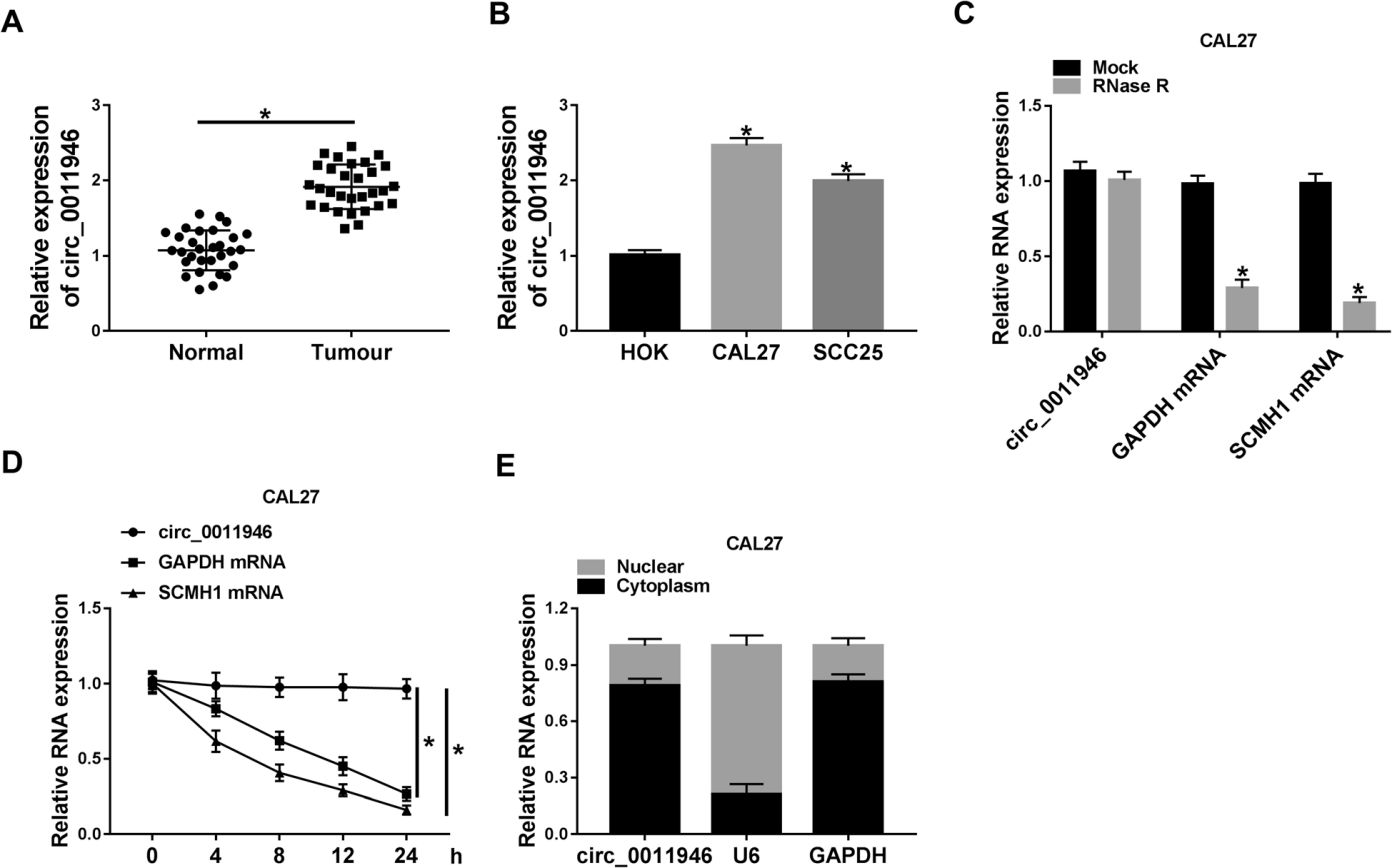
Circ_0011946 expression in OSCC tissues and cells. (**A**) Circ_0011946 level was detected in OSCC and normal tissues. Sample size: *n* = 30. (**B**) Circ_0011946 expression was examined in OSCC cells (CAL27 and SCC25) and HOK cells. (**C**) Circ_0011946, SCMH1 mRNA and GAPDH mRNA levels were detected after treatment of Actinomycin D for different times. (**D**) Circ_0011946, SCMH1 mRNA and GAPDH mRNA levels were measured after treatment of RNase R. (**E**) Subcellular localization assays showing the cytoplasm localization of circ_0011946. ^*^P < 0.05

To analyze whether circ_0011946 was associated with OSCC development, circ_0011946 expression was detected in OSCC tissues and cells. Circ_0011946 level was higher in OSCC tissues compared with normal specimens (Fig. 1A). Moreover, circ_0011946 expression was apparently upraised in CAL27 and SCC25 cells in comparison to HOK cells (Fig. 1B). Furthermore, the ringlike structure of circ_0011946 was certified, since circ_0011946 was resistant to Actinomycin D and RNase R digestion than linear GAPDH and linear SCMH1 (Fig. 1C and D). Additionally, subcellular localization assays showed that circ_0011946 predominantly localized to the cytoplasm (Fig. 1E). Thus, circ_0011946 might contribute to OSCC development.

### Circ_0011946 knockdown constrains the tumorigenic phenotypes of OSCC cells

**Fig. 2:**
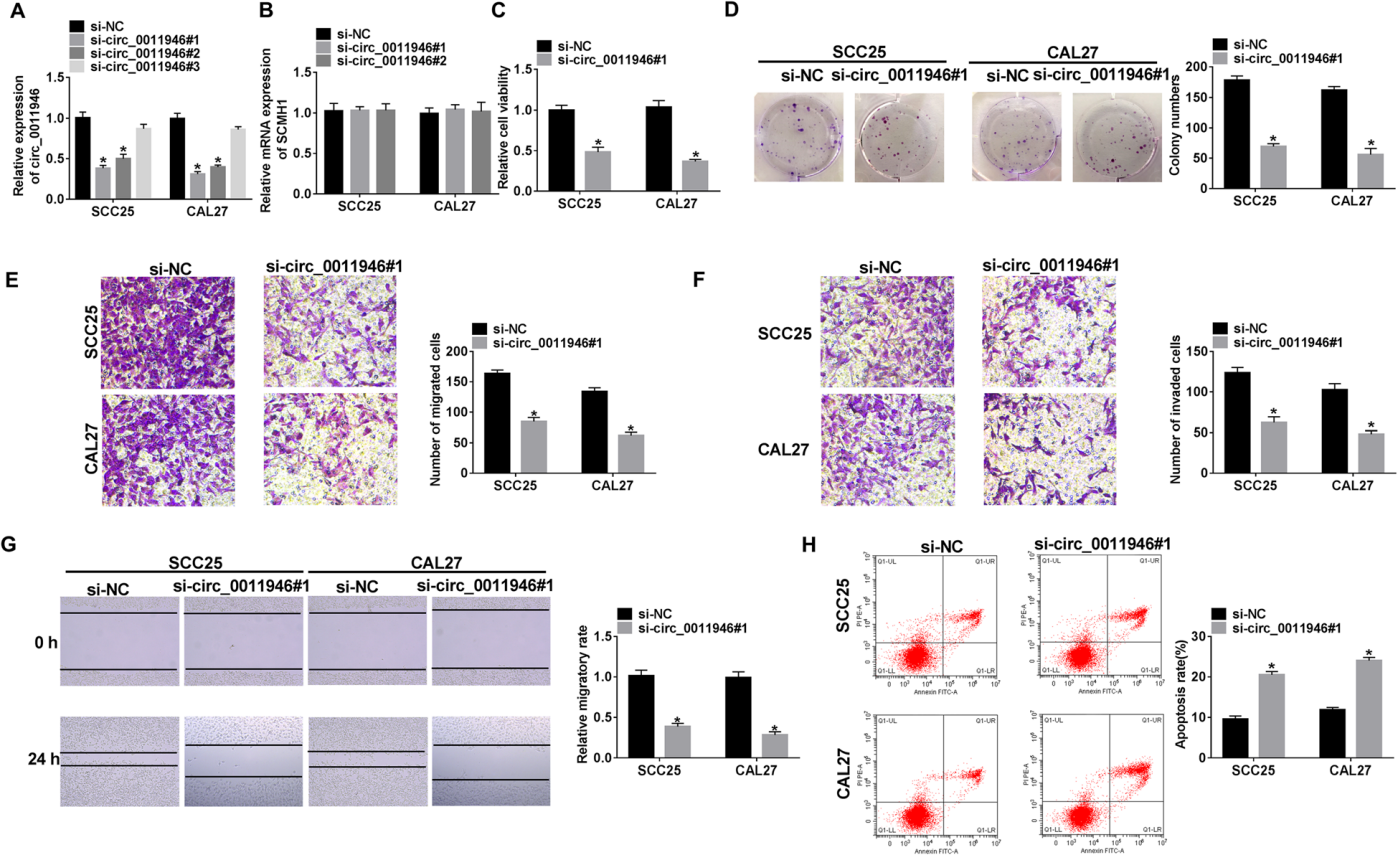
The influence of circ_0011946 on OSCC progression. (**A**) Circ_0011946 level was examined in CAL27 and SCC25 cells with transfection of si-NC, si-circ_0011946#1, #2 or #3. (**B**) SCMH1 expression was detected in CAL27 and SCC25 cells with transfection of si-NC, si-circ_0011946#1 or #2. Cell viability (**C**), colony formation (**D**), migration and invasion (**E**-**G**), and apoptosis (H) were measured in CAL27 and SCC25 cells with transfection of si-NC or si-circ_0011946#1. ^*^P < 0.05

To scrutinize the function of circ_0011946 in OSCC cell progression, siRNAs against circ_0011946 were introduced into CAL27 and SCC25 cells. Transfection of si-circ_0011946#1 and #2 effectively decreased circ_0011946 level, but si-circ_0011946#3 did not affect the abundance (Fig. 2A). Meanwhile, si-circ_0011946#1 and #2 had hardly changed the level of linear SCMH1 (Fig. 2B). Si-circ_0011946#1 with the most remarkable efficacy was chosen for succeeding investigations. Circ_0011946 knockdown markedly decreased cell proliferation by diminishing cell viability and colony formation capability (Fig. 2C and D). Besides, circ_0011946 silence evidently repressed cell migration and invasion (Fig. 2E-G). Likewise, circ_0011946 depletion induced significant apoptosis (Fig. 2H). Hence, circ_0011946 knockdown repressed OSCC cell progression in vitro.

### Circ_0011946 targets miR-216a-5p in OSCC cells

**Fig. 3:**
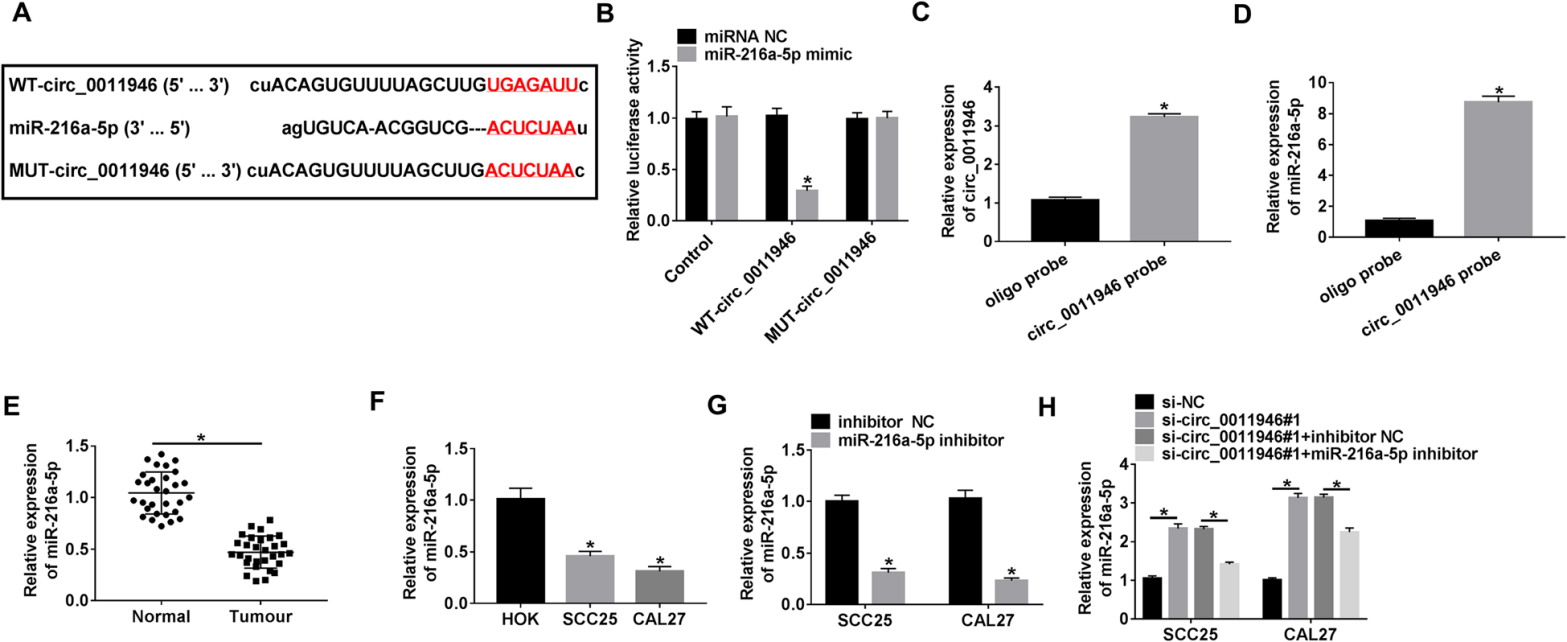
The target relationship between circ_0011946 and miR-216a-5p. (**A**) The binding sequence of circ_0011946 and miR-216a-5p. (**B**) Luciferase activity was measured in CAL27 cells with transfection of control vector, WT-circ_0011946 or MUT-circ_0011946 and miRNA NC or miR-216a-5p mimic. (**C** and **D**) circ_0011946 and miR-216a-5p levels were measured after RNA pull-down using oligo or circ_0011946 probe. (**E**) miR-216a-5p level was determined in OSCC and normal samples. Sample size: *n* = 30. (**F**) miR-216a-5p abundance was examined in OSCC cells (CAL27 and SCC25) and HOK cells. (**G**) miR-216a-5p level was examined in CAL27 and SCC25 cells with transfection of inhibitor NC or miR-216a-5p inhibitor. (**H**) miR-216a-5p abundance was detected in CAL27 and SCC25 cells with transfection of si-NC, si-circ_0011946#1, si-circ_0011946#1 + inhibitor NC or miR-216a-5p inhibitor. ^*^P < 0.05

The target miRNAs of circ_0011946 were searched by starBase database. MiR-216a-5p was targeted by circ_0011946, and the binding site of circ_0011946 and miR-216a-5p was presented in Fig. 3A. MiR-216a-5p overexpression caused a ~ 70% diminution of luciferase activity in WT-circ_0011946 group, even though it makes no difference in the activity in MUT-circ_0011946 group (Fig. 3B). Likewise, RNA pull-down assay disclosed that circ_0011946 and miR-216a-5p were enriched by circ_0011946 probe (Fig. 3C and D). Additionally, miR-216a-5p abundance in OSCC was measured, and reduced miR-216a-5p level was disclosed in OSCC tissue samples and cell lines (Fig. 3E and F). The transfection efficacy of miR-216a-5p inhibitor was identified by qRT-PCR (Fig. 3G). Remarkably, miR-216a-5p level was elevated by circ_0011946 silence, whereas this impact was relieved by miR-216a-5p downregulation (Fig. 3H). Altogether, miR-216a-5p is the target of circ_0011946 in OSCC cells.

### miR-216a-5p downregulation attenuates the influences of si-circ_0011946 on OSCC cell progression

**Fig. 4:**
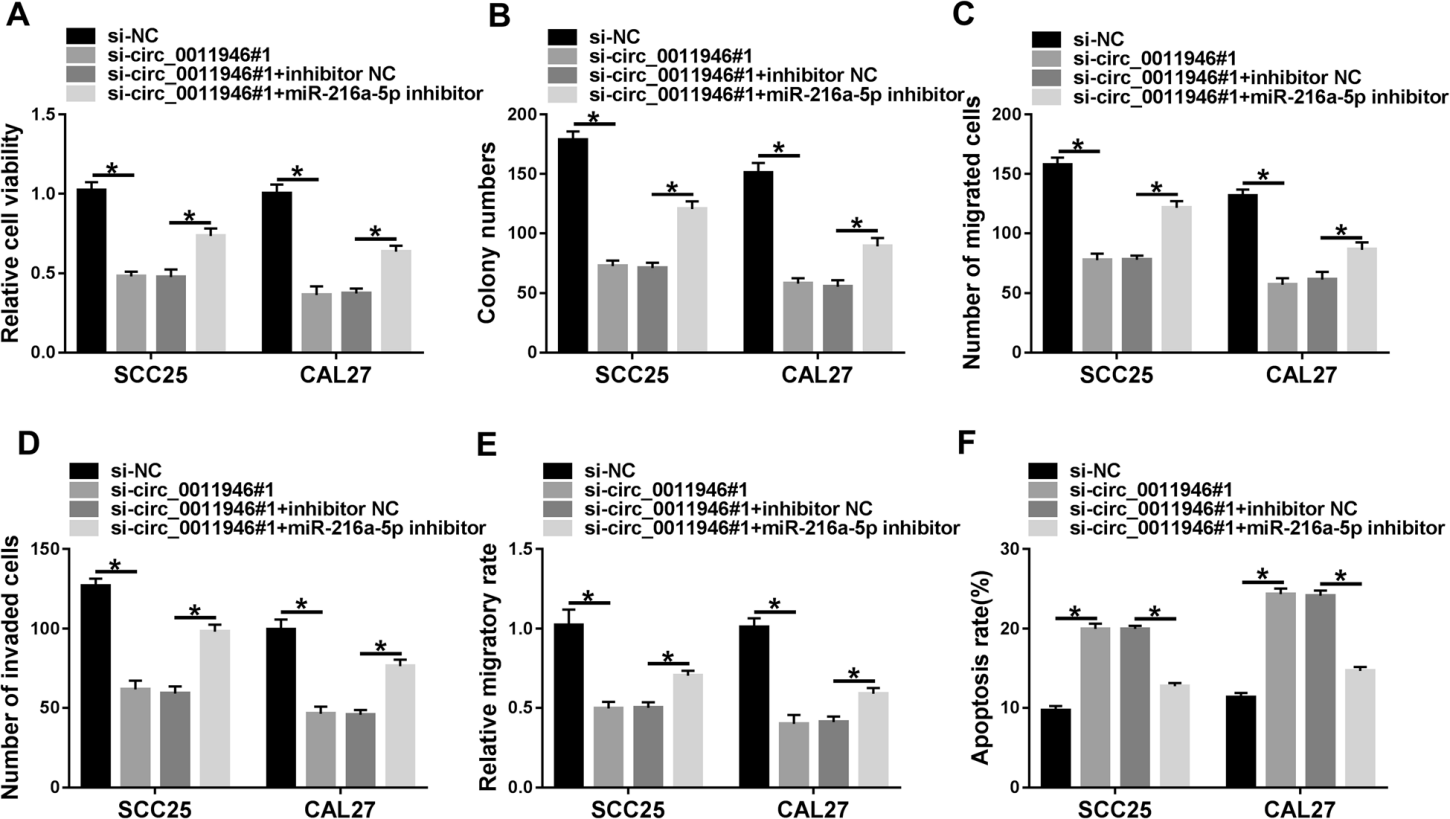
The influence of circ_0011946 and miR-216a-5p on OSCC progression. Cell viability (**A**), colony formation (**B**), migration and invasion (**C**-**E**), and apoptosis (**F**) were examined in CAL27 and SCC25 cells with transfection of si-NC, si-circ_0011946#1, si-circ_0011946#1 + inhibitor NC or miR-216a-5p inhibitor. ^*^P < 0.05

To scrutinize if miR-216a-5p was accountable for the mediation of circ_0011946, CAL27 and SCC25 cells were introduced with si-NC, si-circ_0011946#1, si-circ_0011946#1 + inhibitor NC or si-circ_0011946#1 + miR-216a-5p inhibitor. Indeed, miR-216a-5p reduction mitigated si-circ_0011946-mediated suppression on cell proliferation (Fig. 4A and B). Furthermore, miR-216a-5p knockdown alleviated the inhibitory function of circ_0011946 downregulation on cell migration and invasion (Fig. 4C-E). Additionally, miR-216a-5p deletion diminished circ_0011946 silence-induced cell apoptosis (Fig. 4F). These data suggested that the effects of circ_0011946 silence were due to the up-regulation of miR-216a-5p.

### Circ_0011946 regulates BCL2L2 expression by competitively binding to miR-216a-5p

**Fig. 5:**
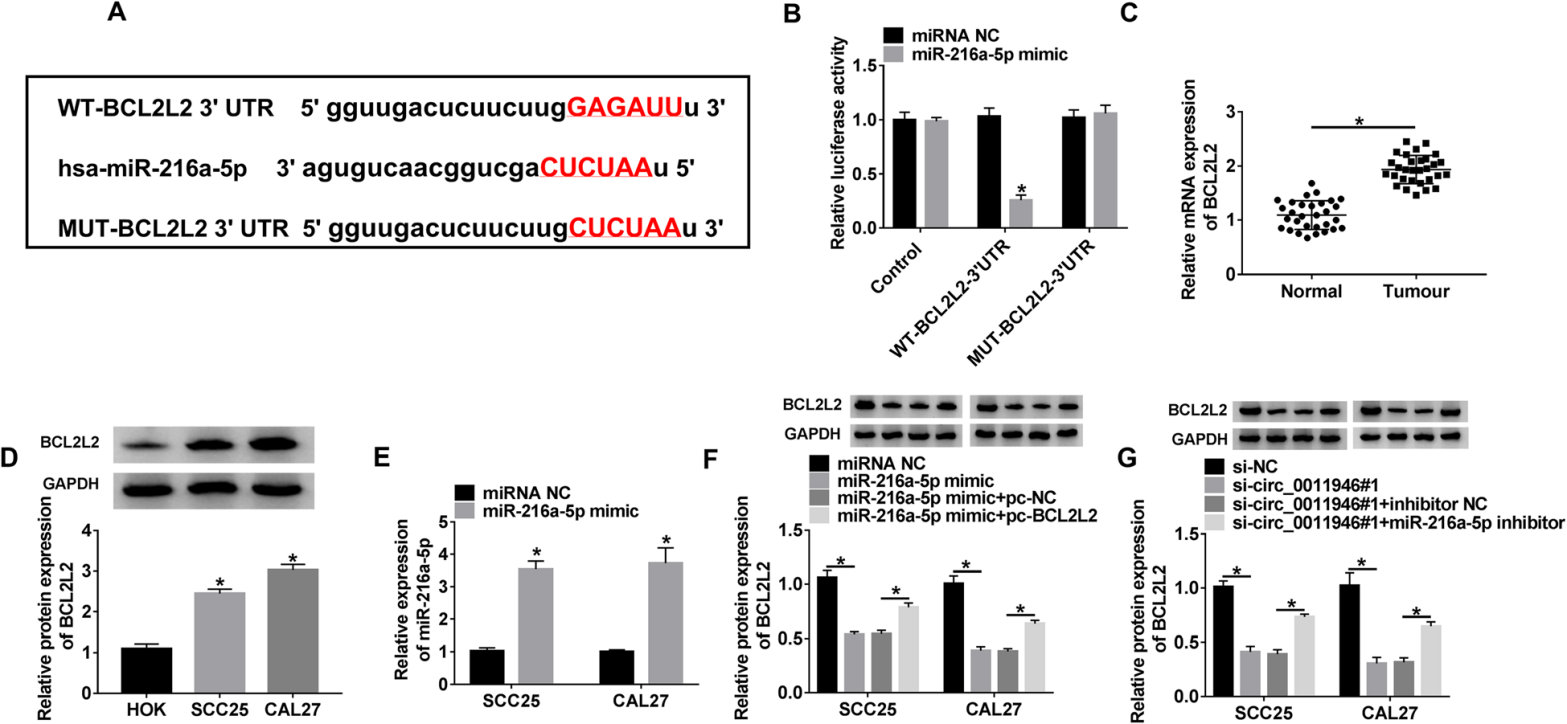
The target relationship between miR-216a-5p and BCL2L2. (**A**) The binding site of miR-216a-5p and BCL2L2. (**B**) Luciferase activity was examined in CAL27 cells with transfection of control vector, WT-BCL2L2–3’UTR or MUT-BCL2L2–3’UTR and miRNA NC or miR-216a-5p mimic. (**C**) BCL2L2 expression was examined in OSCC and normal samples. Sample size: *n* = 30. (**D**) BCL2L2 level was detected in OSCC cells (CAL27 and SCC25) and HOK cells. (**E**) miR-216a-5p abundance was examined in CAL27 and SCC25 cells with transfection of miRNA NC or miR-216a-5p mimic. (**F**) BCL2L2 level was detected in CAL27 and SCC25 cells with transfection of miRNA NC, miR-216a-5p mimic, miR-216a-5p mimic + pc-NC or pc-BCL2L2. (**G**) BCL2L2 abundance was examined in CAL27 and SCC25 cells with transfection of si-NC, si-circ_0011946#1, si-circ_0011946#1 + inhibitor NC or miR-216a-5p inhibitor. ^*^P < 0.05

The molecular targets of miR-216a-5p were forecasted by starBase database, and the data showed that BCL2L2 was a presumed target of miR-216a-5p (Fig. 5A). High level of miR-216a-5p diminished the luciferase activity of WT-BCL2L2–3’UTR by 75%, whereas it exhibited little impact on MUT-BCL2L2–3’UTR group (Fig. 5B). Moreover, BCL2L2 mRNA and protein level were considerably heightened in OSCC tissues and cells (Fig. 5C and D). Additionally, the transfection efficacy of miR-216a-5p mimic was affirmed by qRT-PCR analysis (Fig. 5E). BCL2L2 expression was considerably diminished by miR-216a-5p mimic introduction, which was repaired by transfection of pc-BCL2L2 (Fig. 5F). Additionally, the strong inverse correlation was found between BCL2L2 mRNA and miR-216a-5p expressions in OSCC tissues (Supplement Fig. 1). Furthermore, qRT-PCR results displayed that circ_0011946 silence resulted in decreased expression of BCL2L2 protein, and this influence was revoked by miR-216a-5p knockdown (Fig. 5G). Collectively, circ_0011946 mediated BCL2L2 expression by miR-216a-5p.

### MiR-216a-5p restrains OSCC cell development by managing BCL2L2

**Fig. 6:**
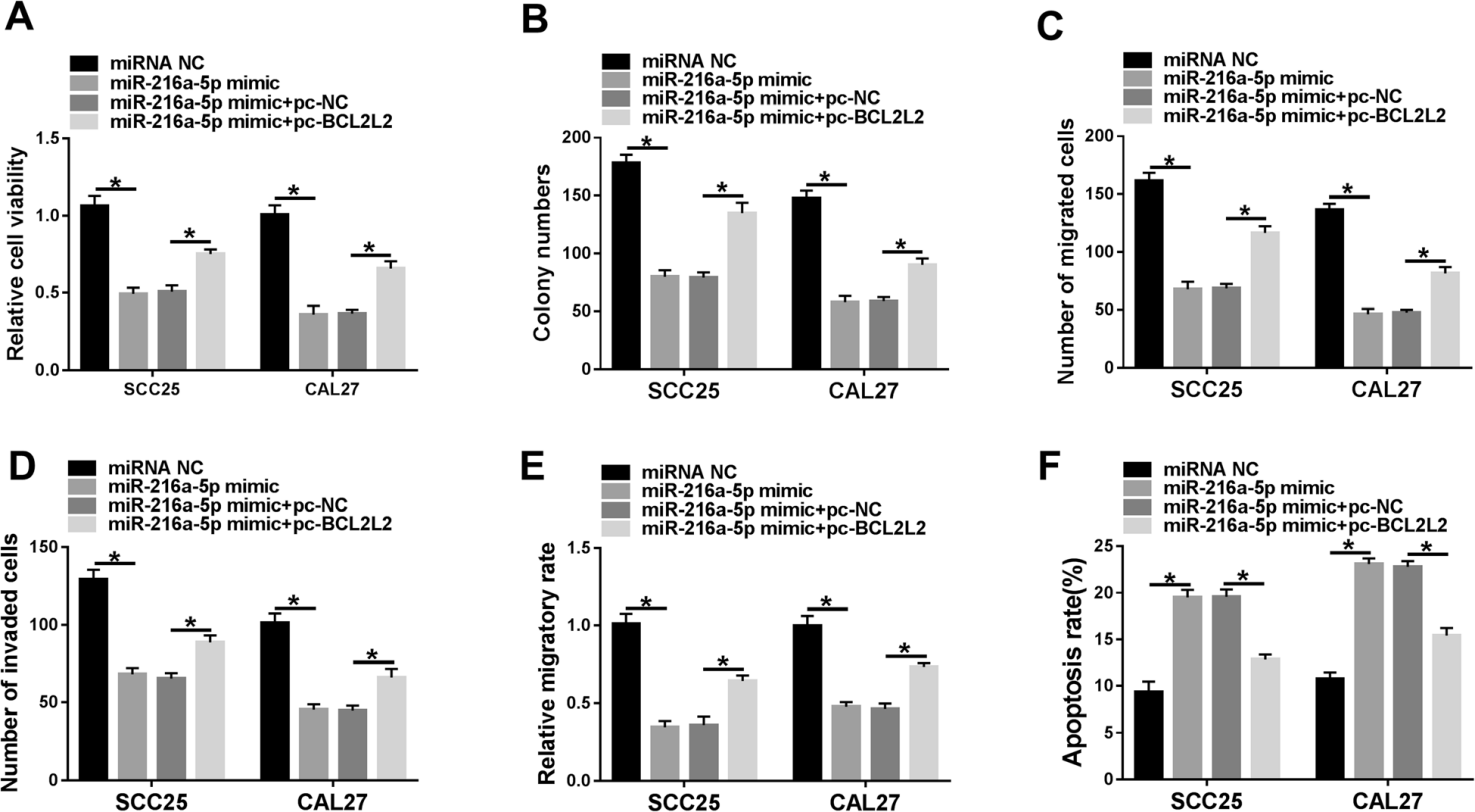
The influence of miR-216a-5p and BCL2L2 on OSCC progression. Cell viability (**A**), colony formation (**B**), migration and invasion (**C**-**E**), and apoptosis (**F**) were examined in CAL27 and SCC25 cells with transfection of miRNA NC, miR-216a-5p mimic, miR-216a-5p mimic + pc-NC or pc-BCL2L2. ^*^P < 0.05

To test the impacts of miR-216a-5p and BCL2L2 in the malignant phenotypes of OSCC, gain-of-function assays were fulfilled in CAL27 and SCC25 cells. Elevated expression of miR-216a-5p obviously constrained cell proliferation, which was reversed by BCL2L2 restoration (Fig. 6A and B). Additionally, miR-216a-5p overexpression impelled a notable inhibition on cell migratory and invasive abilities, and this effect was mitigated by BCL2L2 up-regulation (Fig. 6C-E). Moreover, miR-216a-5p overexpression notably fostered cell apoptosis, which was attenuated by introduction of BCL2L2 expressing plasmid (Fig. 6F). These data uncovered that miR-216a-5p impacted OSCC cell behaviors by regulating BCL2L2.

### Circ_0011946 knockdown reduces tumor growth in xenograft model

**Fig. 7:**
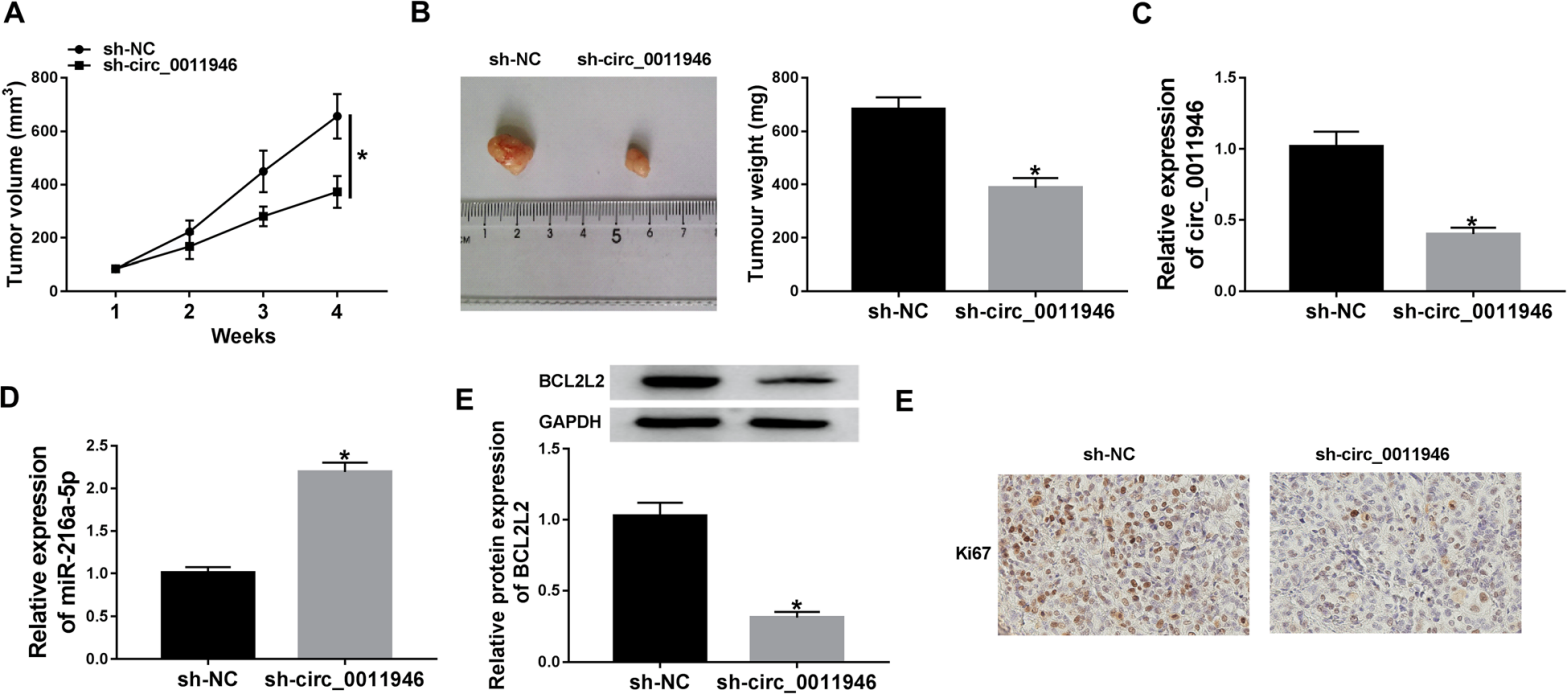
The effect of circ_0011946 on OSCC cell growth in vivo. (**A**) Tumor volume was examined every week. (**B**) Tumor weight was measured at the end point. (**C**-**E**) circ_0011946, miR-216a-5p and BCL2L2 levels were examined in each group. (**E**) Representative images showing Ki67 staining of sections from sh-NC control and sh-circ_0011946 tumors at the end point. ^*^P < 0.05

Subsequently, an in vivo xenograft model was utilized to examine the influence of circ_0011946 in OSCC cell growth. As displayed in Fig. 7A and B, tumor volume and weight of sh-circ_0011946 group were diminished than those in sh-NC group. Besides, circ_0011946 and BCL2L2 levels were lessened in sh-circ_0011946 group as opposed to sh-NC group, while miR-216a-5p level was increased (Fig. 7C-E). Additionally, sh-circ_0011946-transduced tumors had significantly fewer cells stained for Ki67 staining than the sh-NC control (Fig. 7E). These data showed that silencing of circ_0011946 minimized tumor growth in vivo.

## Discussion

OSCC is the commonest type of oral cancers with high morbidity worldwide [18]. Many circRNAs have been revealed to apply important works in tumorigenesis and treatment of OSCC [6]. The objective of this study is to study new mechanism underlying the regulation of circ_0011946 in OSCC.

Meng et al. disclosed that circ_0011946 expression was strengthened in OSCC, and circ_0011946 silence restrained cell proliferation and metastasis in CAL27 cells [9]. Here, circ_0011946 abundance was elevated in OSCC. Moreover, circ_0011946 knockdown could suppress cell proliferation, migration, and invasion, but promote apoptosis in CAL27 and SCC25 cells. This was also in agreement with that in other cancers, such as breast cancer and hepatocellular carcinoma [19, 20]. Additionally, in this study, we used siRNAs targeting circ_0011946 to silence circ_0011946, which are designed by targeting the sequence spanning the junction of circ_0011946. Since the sequence spanning the junction is unique for circ_0011946, the siRNAs do not affect the transcription of the linear mRNA.

Here, we ascertained that miR-216a-5p was sponged by circ_0011946. miR-216a-5p was disclosed to be lower expressed in OSCC, and restrained cell proliferation, migration and invasion [12]. Furthermore, overexpressed miR-216a-5p oppressed OSCC cell development, and its tumor suppressor function was also uncovered in breast cancer, esophageal squamous cell carcinoma, and osteosarcoma [21–24]. Additionally, our findings first demonstrated that the function of circ_0011946 depended, at least in part, on miR-216a-5p.

Next, we confirmed that BCL2L2 was a target and functional target of miR-216a-5p. BCL2L2 has established roles in promoting human tumorigenesis [14, 25–27]. Previous work also reported the oncogenic role of BCL2L2 in OSCC [16]. Moreover, BCL2L2 has not only anti-apoptotic role but also promotes cell migration and invasion [13]. Multiple studies have reported that BCL2L2 enhanced cell migration and invasion in several forms of cancers [15, 28, 29]. Here, we first uncovered that circ_0011946 regulated BCL2L2 level by miR-216a-5p competition. In addition, a xenograft model identified the anti-growth function of circ_0011946 silence in OSCC. However, there was a limitation that we did not analyze the influence of miR-216a-5p/BCL2L2 axis on xenograft tumor growth in vivo, which will be studied in future work.

## Conclusions

Taken together, our findings suggest that circ_0011946 knockdown constrains cell proliferation, invasion and migration, but promotes apoptosis in OSCC at least in part by the miR-216a-5p/BCL2L2 axis.

## Supplementary Information

**Additional file 1: Supplement Figure 1.** Expression correlation between BCL2L2 mRNA and miR-216a-5p levels in OSCC tissues using the Pearson’s rank correlation coefficient.

## Data Availability

All data generated or analysed during this study are included in this published article [and its supplementary information files].

## Abbreviations

circRNAs: circular RNAs
OSCC: oral squamous cell carcinoma
circ_0011946: circRNA hsa_circ_0011946
miR: microRNA
BCL2L2: B cell lymphoma-2-like 2 protein
mTOR: mammalian target of rapamycin
SCMH1: Scm polycomb group protein homolog 1
PCNA: proliferating cells nuclear antigen
miRNAs: microRNAs
EIF4B: eukaryotic translation initiation factor 4B
