# Supplementary figures and images for: Knockdown of circ_0011946 targets miR-216a-5p/BCL2L2 axis to regulate proliferation, migration, invasion and apoptosis of oral squamous cell carcinoma cells
Source: BMC Cancer. 2021 Oct 7;21:1085. doi: 10.1186/s12885-021-08779-4 (PMC8499457; doi:10.1186/s12885-021-08779-4)

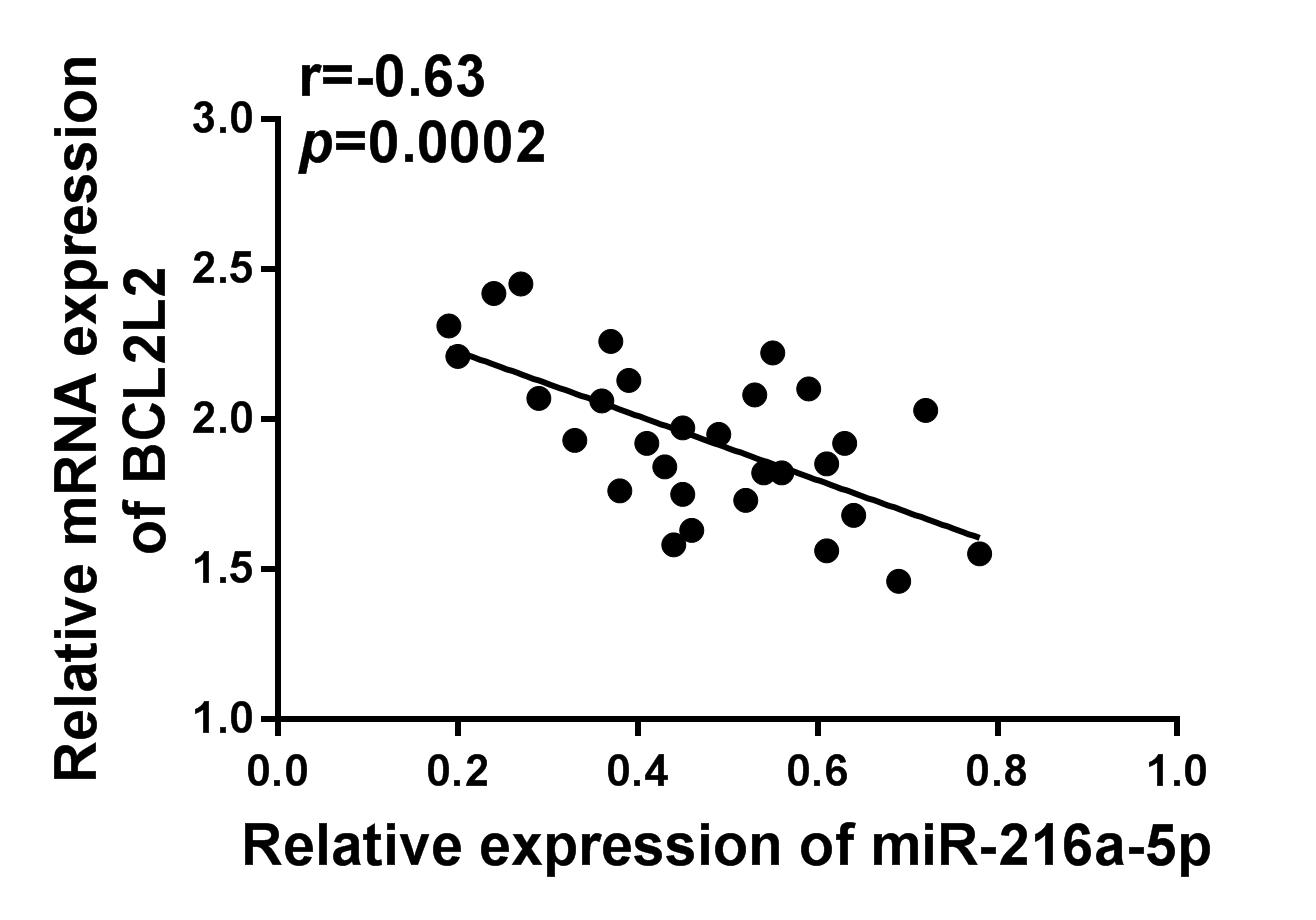

Supplement: Supplementary file 1 — Additional file 1: Supplement Figure 1. Expression correlation between BCL2L2 mRNA and miR-216a-5p levels in OSCC tissues using the Pearson’s rank correlation coefficient. [file 12885_2021_8779_MOESM1_ESM.tif]
